# Agreement between retrospectively assessed health-related quality of life collected 1 week and 12 months post-injury: an observational follow-up study

**DOI:** 10.1186/s12955-019-1139-4

**Published:** 2019-04-23

**Authors:** Juanita Haagsma, Gouke Bonsel, Mariska de Jongh, Suzanne Polinder

**Affiliations:** 1 Department of Public Health, Erasmus MC, University Medical Center Rotterdam, P.O. Box 2040, 3000 CA Rotterdam, The Netherlands; 2 Department Trauma TopCare, ETZ Hospital, Hilvarenbeekseweg 60, 5022 GC Tilburg, The Netherlands

**Keywords:** Health-related quality of life, EQ-5D, Recall bias, Trauma population

## Abstract

**Background:**

Retrospective assessment of pre-injury health-related quality of life (HRQL) is frequently used to measure change from pre- to post-injury HRQL. However, retrospective measurement may be confounded by recall bias. It is assumed that presence of recall bias is influenced by several factors, such as the measurement scale or the instrument that is used, the measurement schedule, and the presence of a substantial health event during the follow up period. This study empirically tests these assumptions by comparing pre-injury EQ-5D summary scores, EQ-5D profiles and visual analogue scale (EQ-VAS) scores of trauma patients, as recorded 1 week and 12 months post-injury, respectively.

**Methods:**

A sample of 5371 adult trauma patients who attended the Emergency Department (ED) followed by hospital admission, received postal questionnaires 1 week (T1) and 12 months (T2) post-injury. The questionnaires contained items on pre-injury health, in terms of EQ-5D3L and EQ-VAS.

**Results:**

One thousand one hundred sixty-six completed data pairs with T1 and T2 pre-injury data were available. Mean pre-injury EQ-5D summary scores were 0.906 (T1) and 0.905 (T2), respectively, with moderate intertemporal agreement (intraclass correlation coefficient (ICC) T1T2 = 0.595). In absolute terms, 442 (37.9%) respondents reported a different pre-injury EQ-5D profile at T2 compared to T1. The least stable EQ-5D dimension was pain/discomfort (20.2% reported a change). Mean T2 pre-injury EQ-VAS score was significantly higher than mean T1 pre-injury EQ-VAS score (T2 84.6 versus T1 83.3). Multivariable logistic regression analysis indicated that lower educational level, comorbid disease and having PTSD symptoms were independent predictors of change of pre-injury EQ-5D profile.

**Conclusions:**

Despite one third of respondents reported a different pre-injury health level, if asked for on two interview occasions separated by 1 year, on the group level this difference was nil (EQ-5D summary score) to small (EQ-VAS). The consistent symmetrical pattern of change suggests random error to play the largest role. Intertemporal reliability was the same in EQ-5D profiles vs. EQ-VAS scores, ruling out scale effects. Particularly certain trauma subgroups showed highest distortion. While group comparisons may be trusted, in pre-post analysis and repeated measure analysis the individual injury impact and recovery pattern may be wrongly estimated.

## Background

Health-related quality of life (HRQL) is a prominent outcome measure in trauma care. HRQL measurement is widely applied in clinical studies, in public health as an estimator of injury impact, as source for patient information, and as indicator (patient reported outcome measurement, PROM) to compare trauma unit performance. For the last purpose, measuring individual change (e.g. from pre- to post-injury HRQL) is critical.

So far two studies obtained prospectively measured pre-injury HRQL and post-injury HRQL, though not intended for performance analysis: the Medical Expenditure Panel Survey (MEPS) [1] and the Seguimiento Universidad de Navarra (SUN) [2] cohort study. However, the impracticability of prospectively collecting HRQL data of trauma patients due to the mere unpredictability of injury events forces clinicians and researchers to rely on retrospective measurement of pre-injury heath as second best. In published studies the period at which pre-injury HRQL is measured (‘retrospective window’) ranges from immediately at admission [3] to 2 years post-injury [4, 5]. Although retrospective assessment of pre-injury HRQL is much easier to implement than a pre-measurement strictu sensu, the measurement is subject to random error and recall bias [6, 7].

We define recall bias as a systematic measurement error, due to selective memory or other content-related reporting effects. The usual direction of recall bias is that – from a current healthy standpoint – poor health in the past is memorized as even more deteriorated as it actually was. The reverse may also happen: from an ill-health standpoint, past health may be memorized better than it actually was. We must distinguish these reporting biases from response shift, which is not a measurement error, but a true change of currently measured outcome due to a (permanent) change of the measurement perspective of the respondent (‘internal measurement scale’), where the usual change is towards adaptation and reduction of cognitive dissonance if present. As a natural consequence patients may both upgrade current impaired health, or downgrade past health. Recall bias causes larger contrasts between healthy pre-state and affected post-state as indicated, while response shift – primarily relevant when health deteriorates – reduces the past-present contrast. Whatever reporting effect may be present, measured pre-post changes may not reflect the true change in HRQL.

Recall bias depends on several factors, some of which are a researcher’s choice. Firstly, recall bias depends on the scale or instrument that is used. Subjective scales, like a visual analogue scale (VAS), may be more easily distort response than a classification or category scale, such as the EQ-5D5L, the Health Utilities Index (HUI), and the Short Form (SF)-36 [8]. This is particularly true if the question phrasing focuses on internal experience or internal values, rather than referring to tangible, observable, labels. Secondly, the measurement schedule matters: the longer the time interval between measurement and situation to be judged, the larger the bias, also because of the increasing risk of a substantial health event, such as a subsequent injury, a medical intervention or deterioration of a pre-existing chronic illness, that may occur between the assessments [6].

Knowledge on recall bias is vital to routine evaluation of injury care. This study aimed to analyze the presence of recall bias, if any, by comparing pre-injury EQ-5D summary scores, EQ-5D profiles and EQ-VAS scores collected shortly after and 12 months after injury.

## Methods

### Study design

This study is linked to the Brabant Injury Outcome Surveillance (BIOS) study. The BIOS study is a prospective observational 12-month follow-up study of trauma patients that has been admitted in one of the 11 hospitals of the Dutch Network Emergency Care Brabant [9]. The BIOS study was approved by the Medical Ethics Committee Brabant, the Netherlands (NL50258.028.14). The BIOS study protocol that was approved included the reported measures and analysis that are presented here.

### Participants

Patients were eligible for inclusion if they met the following inclusion criteria: patients were aged 18 years or older and were seen at the Emergency Department (ED) of one of the 11 hospitals of Network Emergency Care Brabant and were admitted to a ward or an Intensive Care unit (ICU) and survived to hospital discharge. The sample of eligible patients consisted of victims of intentional and unintentional injury, the sustained injuries varied from moderate to severe injury, single and multiple injury. Patients who had a proven pathological (‘spontaneous’) fracture, insufficient knowledge of the Dutch language, or who had no permanent address of residence were excluded. Patients were recruited between March 2016 and November 2016 and were invited to participate at 1 week post-trauma.

Each injury patient that met the inclusion criteria of the study received a postal questionnaire 1 week (T1) and 12 months (T2) after the initial treatment of the injury. It was confirmed that the patient was not deceased before the questionnaire was sent. For these questionnaires the patients needed to give permission by an informed consent form. Informed consent forms were sent together with the T1 questionnaire. A reminder was sent to T1 and T2 non-responders, aiming to increase response rates. A participant number was assigned to the eligible patients that met the inclusion criteria of the study and this number was used to link the T1 and T2 responses.

### Questionnaires

The T1 and T2 questionnaires were strongly related, and included items regarding socio-demographics (age, gender and educational level). Additionally, T1 questionnaire included 21 items regarding the presence of one or more chronic disease(s) prior to the injury to assess comorbidity [10], e.g. chronic non-specific lung disease, heart disease, diabetes, backache, arthrosis, rheumatoid arthritis. Comorbidity was defined as the presence of any co-existing medical condition or disease process additional to the injury that qualified for inclusion [11].

The questionnaires also contained a HRQL module, consisting of the EQ-5D3L classification and the EQ-VAS. The EQ-5D3L classification invites the respondent to assign him/herself to one of three ordinated function levels (grades), in five separate dimensions: mobility, self-care, usual activities, pain/discomfort and anxiety/depression [12, 13]. The T1 and T2 questionnaires included modules for current and for pre-injury EQ-5D3L. By combining the answers of the EQ-5D3L a numerical summary score can be derived called a utility weight. The summary score is computed by a formula that firstly yields a partial weight score for each dimension separately, depending on the reported level for that dimension, and secondly adds these partial utility weights to a score between 0 and 1 commonly. The set of weights per level and per dimension (‘tariff’) has been derived at an earlier stage from preference data of the population [14]. Here the *Dutch tariff* was used [15]. Apart from the classification, the HRQOL-modules contained the EQ-5D VAS. The EQ-VAS score ranges from 0 to 100, indicating the “the worst health you can imagine” to “the best health you can imagine”.

### Injury data

For our study cohort, injury related characteristics, including the Abbreviated Injury Scale (AIS) [16] and the Injury Severity Score (ISS) [17], were already available from the Brabant Trauma Registry. The ISS is a scoring system that provides an overall score for patients with multiple injuries that ranges from 0 (no injury) to 75. The ISS is calculated by first determining the highest AIS severity code in each of the three most severely injured ISS body regions (head or neck including cervical spine, face, chest, abdomen or pelvic contents, extremities or pelvic girdle and external) and subsequently squaring and summing the numbers of these AIS severity codes. Generally, an ISS score ≥ 16 is considered major trauma [18].

### Posttraumatic stress symptoms

Posttraumatic stress disorder (PTSD) is highly prevalent in trauma populations and according to the *Diagnostic and Statistical Manual of Mental Disorders (DSM) IV* memory disturbances are part of the diagnostic criteria of PTSD [19]. Therefore, in our study PTSD symptoms were included as a determinant of memory problems. In our study the impact of event scale (IES) was used to assess symptoms of posttraumatic stress indicative of PTSD 12 months post-injury [20]. The IES consists of 15 items, which measure intrusive re-experiences of the trauma and avoidance of trauma-related stimuli. From the responses on the 15 items the total IES-score, ranging from 0 through 75, can be calculated. Wohlfarth et al. showed that, if 35 points are chosen as cut-off score, and if the multi-criteria PTSD diagnosis of the DSM-IV was chosen as verified outcome, that the sensitivity was 0.89, and the specificity was 0.94 [21]. We adhered to this 35 cut off point. The Dutch translation of the IES has been found to be valid and reliable [22].

### Hypotheses

We formulated four hypotheses:

- Recalled pre-injury HRQL – assumed to be a healthy state, which cannot change ex post – is reported worse with increasing retrospective window, due to recall bias.
- The gap between T1 and T2 pre-injury HRQL is larger among severely injured patients (ISS ≥ 16): the cognitive dissonance effect is stronger because particularly in this group of patients rehabilitation is long and patients adapt to their non-optimal post-state.
- The gap between T1 and T2 pre-injury HRQL is larger among patients with PTSD, because this group is presumed to be affected by memory disturbances due to PTSD.
- Agreement between T1 and T2 pre-injury EQ-VAS is lower compared to T1 and T2 agreement in EQ-5D3L terms, as EQ-VAS is more subjective and thus more prone to recall bias.

The first hypothesis is related to the effect of the measurement schedule on recall bias, whereas the second and third hypotheses are related to effect of high impact events and their direct or indirect effect on recall bias. The last hypothesis is related to the effect of the measurement scale on recall bias.

### Statistical analysis

For analysis of the data the Statistical Package for the Social Sciences version 23 was used (SPSS Inc., Chicago, Ill). Chi-square statistics (dichotomous variables) and Fisher’s exact tests were used to test for differences between the non-respondents and the respondents. Non-parametric variables (age) were tested using the Mann–Whitney U-test.

For the analysis only complete pairs of T1-T2 responses were selected. Independent t-tests were used to compare subgroups within the T1 responses (EQ-5D summary scores, EQ-VAS scores): males vs. females, those aged < 65 vs. ≥65 years, absent vs. present pre-existing comorbidity, ISS < 16 vs. ISS ≥ 16, and absence vs. presence of PTSD. One-way *analysis* of variance (*ANOVA*) was *used* to compare EQ-5D-summary and EQ-VAS scores on T1, according to educational attainment (three levels). We compared T1 with T2 group wise, to test the hypotheses on the size of the retrospective window, the injury severity and PTSD, with p*aired* t-tests. We compared T1-T2 EQ-5D summary scores and EQ-VAS scores for all respondents and for subgroups (gender, age, educational level, comorbidity status, injury severity level and PTSD status).

*With the* intraclass correlation coefficient (ICC) we tested the similarity of the T1 and T2 scores for pre-injury EQ-5D summary score and EQ-VAS score. The ICC both catches intercept and constant effects between T1 and T2. We constructed Bland-Altman plots with limits of agreement to visually represent agreement between T1 and T1 EQ-5D and EQ-VAS. We also investigated differences between T1 and T2 pre-injury EQ-5D profiles with some detail, by dichotomizing the T1-T2 change: any level change was assigned score 1, otherwise it was 0. We finally used the five level indicators of the EQ-5D as numerical variable, resulting in a simple profile sum. The change of this sum was used to compare T2 with T1, which assumes that an upward level change in one domain may compensate a downward level change on another domain (levels 1/0/− 1).

We predicted a change, either increase or decrease, in pre-injury EQ-5D profile and EQ-VAS (yes or no) from the socio-demographic factors, injury severity level (ISS < 16 and ISS ≥ 16) and PTSD status (yes or no). Univariate and multivariable logistic regression analysis was applied, with backward deletion (deselection criterion *p* < 0.05).

Overall *P*-values< 0.05 were considered to indicate statistical significance.

## Results

### Study population

The response rate of the first follow-up survey (T1) was 26.5% (*n* = 1518), of which 759 (50.0%) completed the survey within two weeks of sustaining the injury. The response rate of the 12-month follow-up survey (T2) was 82.7% (*n* = 1255). The T1 and T2 pre-injury EQ-5D was completed by 1166 persons. Respondents were significantly younger than non-respondents (median age 62 versus median age 66, *p* < 0.05), the proportion males was higher (non-responders: 53% male versus responders: 46% males, *p* < 0.05).

### EQ-5D summary scores

Group wise comparisons *–* Mean EQ-5D summary scores at T1 and T2 were 0.906 and 0.905 respectively (see Table 1). Lower pre-injury EQ-5D summary score was associated with being female, being older, lower educational level and the presence of pre-existing comorbidity (see Table 1). There was no difference in mean T1 and T2 pre-injury EQ-5D summary scores, except for patients without pre-existing morbidity. This group of patients had significantly lower recalled pre-injury EQ-5D scores at T2, compared to T1 (*p* < 0.05).

**Table 1:** Mean pre-injury EQ-5D summary score assessed 1 week (T1) and 12 months (T2) post-injury and intraclass correlation coefficients (ICC)

	n	Pre-injury EQ-5D assessed at T1 (1 week post-injury)	Pre-injury EQ-5D assessed at T2 (12 months post-injury)	*P*-value	ICC (95% CI)
Total	1166^$^	0.906 (SD 0.17)	0.905 (SD 0.17)	0.886	0.595 (0.56, 0.63)
Gender					
Males	615	0.930 (SD 0.14)	0.922 (SD 0.16)	0.208	0.540 (0.48, 0.59)
Females	551	0.879 (SD 0.18)	0.886 (SD 0.18)	0.324	0.626 (0.57, 0.67)
Age					
< 65 years	575	0.940 (SD 0.12)	0.932 (SD 0.16)	0.157	0.525 (0.46, 0.58)
≥ 65 years	591	0.872 (SD 0.19)	0.879 (SD 0.18)	0.343	0.651 (0.56, 0.66)
Educational level					
Low	309	0.849 (SD 0.22)	0.862 (SD 0.20)	0.242	0.551 (0.47, 0.62)
Middle	450	0.916 (SD 0.15)	0.914 (SD 0.17)	0.760	0.640 (0.58, 0.69)
High	380	0.943 (SD 0.11)	0.936 (SD 0.14)	0.217	0.539 (0.46, 0.61)
Unknown	27	0.851 (SD 0.15)	0.809 (SD 0.20)	0.214	0.535 (0.21, 0.76)
Comorbidity status					
No comorbidity	498	0.975 (SD 0.08)	0.960 (SD 0.11)	0.003*	0.408 (0.33, 0.48)
Comorbidity	649	0.851 (SD 0.19)	0.862 (SD 0.20)	0.138	0.575 (0.52, 0.63)
Unknown	19	0.955 (SD 0.09)	0.919 (SD 0.13)	0.152	0.505 (0.10, 0.77)
Injury Severity Score					
ISS < 16	1093	0.905 (SD 0.17)	0.904 (SD 0.17)	0.907	0.590 (0.55, 0.63)
ISS > =16	65	0.928 (SD 0.16)	0.924 (SD 0.19)	0.813	0.636 (0.47, 0.76)
Unknown	8	0.819 (SD 0.13)	0.820 (SD 0.18)	0.980	0.825 (0.35, 0.96)
PTSD status^&^					
No PTSD (IES < 35)	696	0.927 (SD 0.14)	0.932 (SD 0.13)	0.249	0.564 (0.51, 0.61)
PTSD (IES > =35)	121	0.829 (SD 0.23)	0.816 (SD 0.25)	0.514	0.582 (0.45, 0.69)
Unknown	349	0.891 (SD 0.18)	0.882 (SD 0.20)	0.325	0.592 (0.52, 0.66)

*SD* standard deviation, *ICC* Intraclass correlation coefficient, *ISS* injury severity score

$ Patients who completed the pre-injury EQ-5D shortly after and 12 months after sustaining an injury

& PTSD status was measured with the Impact of Event Scale (IES) 12 months post-injury

*p < 0.05

**Fig. 1:**
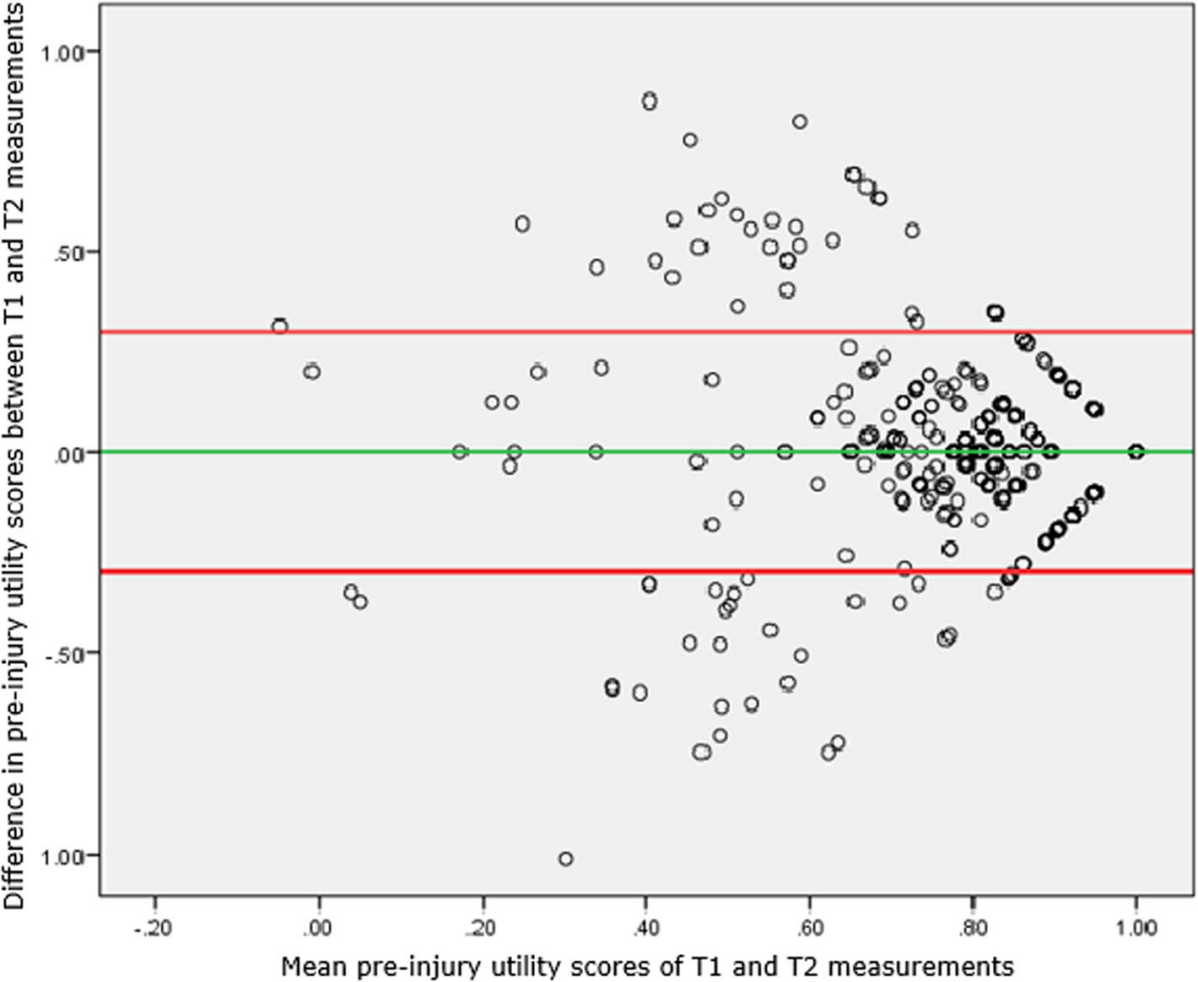
Bland-Altman plot for agreement in T1 and T2 pre-injury EQ-5D utility scores

Pairwise comparisons *–* Pre-injury EQ-5D summary scores measured at T1 and T2 were moderately similar (ICC = 0.595). This was consistent with the Bland-Altman plot in Fig. 1. Figure 1 shows the differences between the two pre-injury EQ-5D summary scores plotted against the averages of the two pre-injury EQ-5D summary scores. For example, if a patient has T1 and T2 pre-injury EQ-5D summary scores of 0.89 and 0.81, respectively, than the mean T1-T2 pre-injury utility scores is 0.85 and the difference in T1-T2 pre-injury utility scores is 0.08. The Bland-Altman plot in Fig. 1 covers a moderate proportion of the possible change scores. The difference between T1 and T2 utility scores is largest when an average T1-T2 utility score is between 0.4 and 0.8.

The inter-temporal agreement between the T1 and T2 scores was lowest in the subgroup no pre-existing morbidity (ICC = 0.408) and highest in the subgroup ≥65 years (ICC = 0.651) and patients with unknown ISS (ICC = 0.825).

### Pre-injury EQ-5D profile

Fifty two out of 243 (21.4%) possible EQ-5D profiles were reported at T1 and T2. An optimal health profile 11111 was reported by 727 patients (62.3%) at T1 and 757 patients (64.9%) at T2.

In total, 724 (62.1%) of 1166 respondents had identical EQ-5D profiles at T1 and T2 (see Table 2). Of these, the majority (*n* = 634; 87.6%) had the optimal health profile 11111. Of the changed respondents, only 93 (21.0%) had a T1 profile of 11111. Half of the changed respondents reported more, the other half reported less problems at T2, indicating random error. Twenty seven respondents (2.3%) reported a different pre-injury EQ-5D profile at T1 and T2, but still had a similar T1 and T2 summary score. The EQ-5D dimensions that differed most frequently between T1 and T2 were pain/discomfort (20.2% of the respondents with a change), mobility (13.1%) and daily activities (13.0%).

**Table 2:** Correspondence of pre-injury EQ-5D profile assessed 1 week (T1) and 12 months (T2) post-injury

Pre-injury EQ-5D	n	T1 = T2	T1 < T2	T1 > T2
EQ-5D profile	1166	62.1%^a^	18.2%^a^	18.3%^a^
Mobility	1166	86.9%	6.2%	6.9%
Self-care	1166	93.0%	3.4%	3.6%
Usual activities	1166	87.0%	6.8%	6.2%
Pain/discomfort	1166	79.8%	9.5%	10.7%
Anxiety/depression	1166	90.1%	6.2%	3.8%

T1 = T2: respondents filled in exactly the same pre-injury EQ-5D profile at T1 and T2

T1 < T2: respondents that reported less problems with the EQ-5D at T2

T1 > T2: respondents that reported more problems with the EQ-5D at T2

^a^does not add up to 100% (2.3% of respondents reported a different EQ-5D profile at T1 and T2, but the profile summary score of the profiles were the same)

### Pre-injury EQ-VAS score

Group wise comparisons *–* Mean EQ-VAS scores at T1 and T2 were 82.9 and 84.4, respectively (see Table 3). Lower pre-injury EQ-VAS scores were associated with being female, being older, lower educational level, the presence of pre-existing comorbidity and having PTSD symptoms indicative of PTSD (see Table 3). The T2 pre-injury EQ-VAS scores were significantly higher than the T1 pre-injury VAS scores. This was true for each sociodemographic subgroup of patients, but the difference was only significant for subgroups females, patients aged 65 and older, and patients with lower and middle educational level. Patients with less injury impact (ISS < 16, PTSD absent) showed considerably larger T1-T2 gap where T1 was worse.

**Table 3:** Mean pre-injury VAS scores assessed 1 week (T1) and 12 months (T2) post-injury and intraclass correlation coefficients (ICC)

	n	Pre-injury EQ-VAS assessed at T1 (1 week post-injury)	Pre-injury EQ-VAS assessed at T2 (12 months post-injury)	*P*-value	ICC
Total	1132	82.9 (SD 15)	84.4 (SD 14)	0.001*	0.580 (0.54, 0.62)
Gender					
Males	596	85.1 (SD 14)	85.8 (SD 13)	0.191	0.580 (0.53, 0.63)
Females	536	81.3 (SD 16)	83.3 (SD 15)	0.001*	0.571 (0.51, 0.63)
Age					
< 65 years	564	86.4 (SD 13)	86.9 (SD 14)	0.324	0.564 (0.51, 0.62)
65+ years	568	80.3 (SD 16)	82.4 (SD 15)	0.000*	0.565 (0.51, 0.62)
Educational level					
Low	295	79.1 (SD 16)	81.4 (SD 15)	0.007*	0.548 (0.47, 0.62)
Middle	439	84.2 (SD 16)	85.7 (SD 14)	0.021*	0.603 (0.54, 0.66)
High	373	86.3 (SD 12)	86.6 (SD 12)	0.582	0.585 (0.52, 0.65)
Unknown	25	74.0 (SD 16)	75.2 (SD 20)	0.798	0.142 (−0.24, 0.48)
Comorbidity status					
No comorbidity	486	89.7 (SD 12)	90.0 (SD 10)	0.168	0.423 (0.35, 0.49)
Comorbidity	628	78.4 (SD 15)	80.4 (SD 16)	0.083	0.557 (0.50, 061)
Unknown	18	84.9 (SD 11)	87.4 (SD 12)	0.111	0.353 (−0.09, 0.69)
Injury Severity Score					
ISS < 16	1060	83.2 (SD 15)	84.6 (SD 14)	0.001*	0.582 (0.54, 0.62)
ISS > =16	64	87.4 (SD 13)	87.4 (SD 14)	0.991	0.659 (0.50, 0.78)
Unknown	8	69.4 (SD 23)	69.6 (SD 27)	0.979	0.448 (−0.31, 0.86)
PTSD symptoms^&^					
No PTSD (IES < 35)	629	84.9 (SD 13)	86.1 (SD 12)	0.003*	0.655 (0.61–0.70)
PTSD (IES > =35)	111	78.4 (SD 17)	79.0 (SD 19)	0.869	0.495 (0.35, 0.62)
Unknown	320	81.5 (SD 17)	83.5 (SD 15)	0.022*	0.515 (0.43, 0.59).

*SD* standard deviation, *ICC* Intraclass correlation coefficient, *ISS* injury severity score

^&^PTSD symptoms measured with the Impact of Event Scale (IES) 12 months post-injury

*p < 0.05

An EQ-VAS score of 100 was reported by 182 patients (16.1%) at T1 and 177 patients (15.6%) at T2. Of these patients, respectively 92 and 89 reported an EQ-5D of 11111. Approximately one in seven patients (*n* = 172, 15.2%) had a difference of 10 points or more between the T1 and T2 pre-injury EQ-VAS score in either direction.

**Fig. 2:**
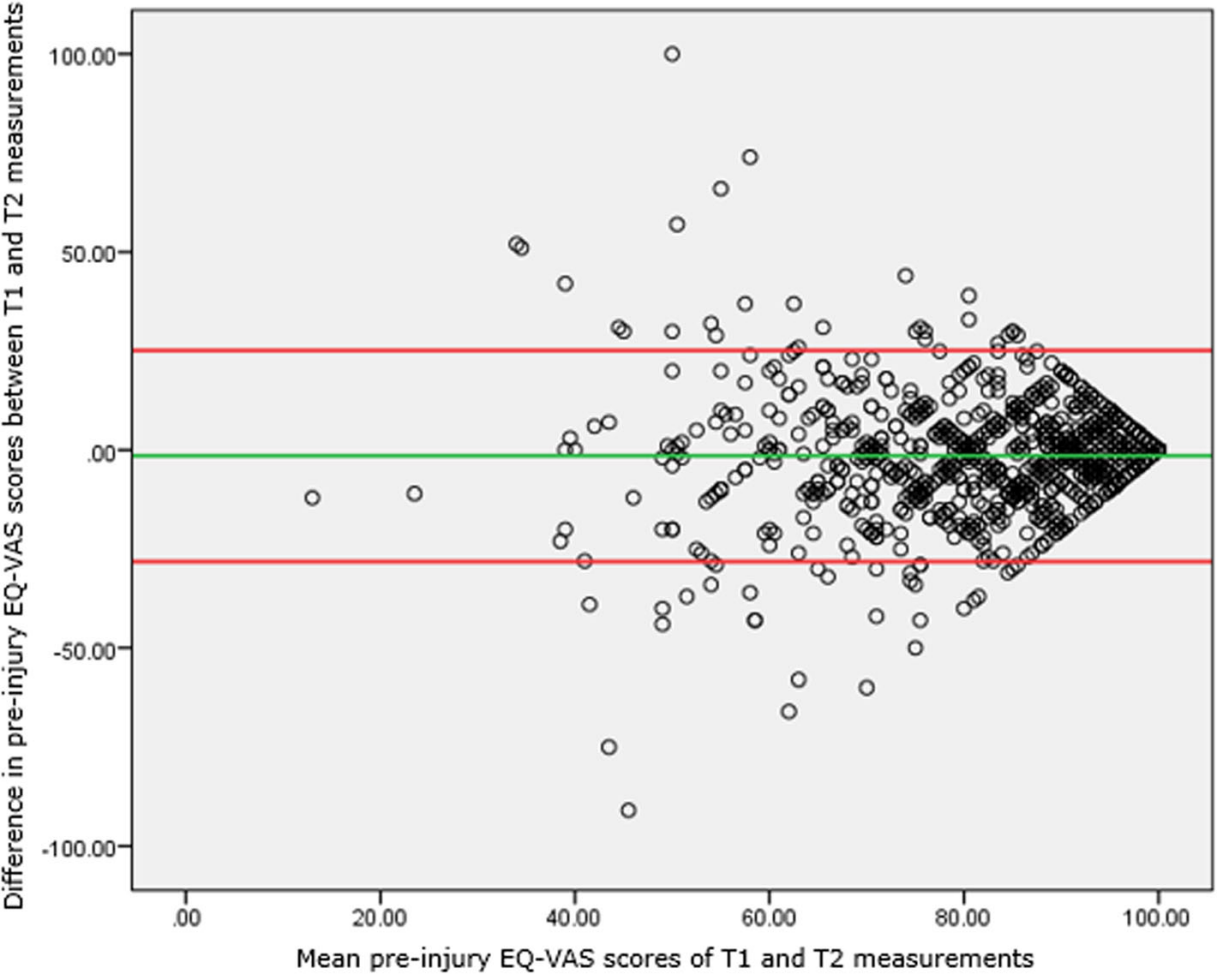
Bland-Altman plot for agreement in T1 and T2 pre-injury EQ-VAS scores

Pairwise comparisons *–* Pre-injury EQ-VAS scores at T1 and T2 corresponded moderately (ICC = 0.580). This was visualized with the Bland-Altman plot in Fig. 2. Figure 2 shows the differences between the two pre-injury EQ-VAS scores plotted against the averages of the two pre-injury EQ-VAS scores. The Bland-Altman plot in Fig. 2 covered a moderate proportion of the possible change scores.

### Factors associated with change in pre-injury EQ-5D profile and EQ-VAS score

EQ-5D profile – Univariate logistic regression analyses showed that females, older age, lower educational level, having co-morbid disease and having PTSD were significantly associated with a change, either increase or decrease, of EQ-5D profile at T1 versus T2. Multivariable logistic regression analysis indicated that having comorbid disease (odds ratio (OR) 3.3, 95% CI 2.3–4.7), having PTSD symptoms indicative of PTSD (OR 2.3; 95% CI, 1.5 to 3.6) and increasing age (OR 1.02; 95% CI, 1.01 to 1.03) are independent factors that are associated with a change of T2 EQ-5D profile compared to T1.

EQ-VAS – Univariate logistic regression analyses showed that none of the factors were significantly associated with a difference of > 1 EQ-VAS point between T1 and T2.

## Discussion

### Main findings

Our study showed that pre-injury health status as measured with EQ-5D3L showed expected patterns according to e.g. age and morbidity. A larger retrospective window (1 year vs 1 week) generally did not systematically influence the reported HRQL levels. Pre-injury health status as measured with EQ-5D3L one week versus one year after the injury did not show any systematic difference on the group level. It showed, however, that a substantial number of respondents showed small changes of health profile or EQ-VAS, in both directions. The consistent symmetrical pattern of change suggests that changes predominantly are the result of random error, rather than bias. The factors associated with change into *any* direction (older age, co-morbid disease, PTSD) may be factors associated with precision of these measures as such.

The least stable EQ-5D dimension between T1 and T2 was pain/discomfort. This may be due to the fact that pain/discomfort is a more subjective dimension compared to mobility, self-care and usual activities [23]. However, anxiety/depression, which is also a more subjective dimension of the EQ-5D, was much more stable between T1 and T2, whereas mobility and usual activities also differed frequently between T1 and T2.

The EQ-5D summary scores and EQ-VAS scores of respondents with severe (ISS > 16) injuries and respondents with PTSD did not differ significantly. Kwong et al. argued that, compared to continuous measures, the more restricted range of responses of generic measures, such as the EQ-5D-3 L, may lead to smaller variability in scores [24]. In our study this may have led to greater homogeneity in pre-injury EQ-5D assessed at T1 and T2 compared to pre-injury EQ-VAS. However, our findings also show that pre-injury EQ-5D was just as easily distorted as the EQ-VAS. More than one in three respondents reported a different pre-injury EQ-5D health state at T1 compared to T2, whereas only one in eight reported a different pre-injury EQ-VAS score at T1 compared to T2.

### Comparison to previous studies

Many studies have retrospectively collected pre-injury HRQL [25]. However, to our knowledge none of these studies have assessed pre-injury HRQL at multiple points in time post-injury. Litwin and McGuigan (1999) did compare retrospectively collected health status data at multiple follow up assessments. In their study Litwin and McGuigan investigated agreement of pre-injury HRQL in men who had undergone radical prostatectomy for early-stage prostate cancer using the RAND 12-Item Short-Form Health Survey (SF-12) [26]. No differences between recollected pre-event SF-12 HRQL between 7 and 37 months port-surgery were found. Howell et al. (2008) who investigated retrospectively collected health status after total hip replacement also did not find systematic differences between recalled pre-event SF-12 between 3 days and 3 months after surgery [27]. This is in agreement with the findings of our study, even though our study differed with regards to several factors, such as difference in time period of pre-event HRQL assessments, difference in the event, difference in instrument that was used to assess pre-event HRQL, and difference in patient groups that were investigated. Each of these factors may affect agreement between retrospectively assessed pre-event HRQL measurements [8, 28].

### Strengths and limitations

This study had several strengths. First, the number of respondents was high and it was therefore possible to test for differences within specific subgroups, such as patients with pre-existing comorbidity and patients who sustained severe injuries. Second, this study assessed both pre-injury EQ-VAS and pre-injury EQ-5D which allowed for comparisons of pre-injury HRQL over time on a subjective scale and a classification like scale. Third, the time period between the two follow up moments was almost one year. With this long time period between measurements it is not very likely that a respondent remembered exactly what he/she filled in at T1. A second advantage of this long time period between measurements is that it allowed us to study recall of pre-event HRQL shortly after and long after injury. The time period between the event and measurement of recalled pre-event HRQL is identified as one of the factors influencing patient recall and it is assumed that asking for a recall over longer periods leads to more recall bias [6].

A limitation of this study was the low rate of patients that responded to the surveys and that filled in the EQ-5D at both follow up moments. The low response rate in our survey may have resulted from the length of the questionnaires, which included items on pre-injury and current health as well as health care consumption and return to work. It is likely that patients with limited interest in these aspects or patients with cognitive impairments were less likely to respond. As a result, the respondents that have filled out the questionnaires may have been a biased and self-selected sample with higher consistency between two measurements and this may have affected the internal validity of our findings. Moreover, the low and possible biased sample may affect the generalizability of our results to the Dutch trauma population.

A second limitation is that we administered a stand-alone paper-and-pencil questionnaire, which did not allow us to verify if the respondents understood the HRQL questions. Our results showed that half of the respondents who reported full health on the EQ-VAS also reported one or more problems on the EQ-5D. This seems contrary to what is expected and may indicate that some of the respondents did not understand the pre-injury EQ-VAS question and/or pre-injury EQ-5D questions. Nonetheless, the findings that the mean pre-injury EQ-5D summary and EQ-VAS scores were lower for females, older respondents, respondents with pre-existing comorbidity, and patients with lower educational level corresponds to findings from other studies on HRQL [29–31]. Therefore, we assume that only a small proportion of the respondents may have had difficulty filling the pre-injury EQ-VAS question and that this had only a small effect on our findings.

## Conclusions

Despite one third of respondents reported a different pre-injury health level, if asked for on two interview occasions separated by 1 year, on the group level this difference was nil (EQ summary score) to small (EQ-VAS). The consistent symmetrical pattern of change suggests that random error plays the largest role. Intertemporal reliability was the same in EQ-5D profiles vs. EQ-VAS scores, ruling out scale effects. Particularly certain trauma subgroups showed highest distortion. While group comparisons may be trusted, in pre-post analysis and repeated measure analysis the individual injury impact and recovery pattern may be wrongly estimated.

## Abbreviations

AIS: Abbreviated Injury Scale
ANOVA: One-way *analysis* of variance
BIOS: Brabant Injury Outcome Surveillance
*DSM-IV*: *Diagnostic and Statistical Manual of Mental Disorders IV*
ED: Emergency Department
HRQL: Health-related quality of life
HUI: Health Utilities Index
ICC: Intraclass correlation coefficient
ICU: Intensive Care unit
IES: Impact of event scale
ISS: Injury Severity Score
MEPS: Medical Expenditure Panel Survey
OR: Odds ratio
PTSD: Posttraumatic stress disorder
RAND 12: Short-Form Health Survey-12
SF-36: Short Form-36
SUN: Seguimiento Universidad de Navarra
T1: Postal questionnaire 1 week after the initial treatment of the injury
T1: Postal questionnaire 12 months after the initial treatment of the injury
VAS: Visual analogue scale
